# Establishment of environmentally sensitive DNA methylation states in the very early human embryo

**DOI:** 10.1126/sciadv.aat2624

**Published:** 2018-07-11

**Authors:** Noah J. Kessler, Robert A. Waterland, Andrew M. Prentice, Matt J. Silver

**Affiliations:** 1 Medical Research Council Unit The Gambia at the London School of Hygiene and Tropical Medicine, London WC1E 7HT, UK.; 2 U.S. Department of Agriculture/Agricultural Research Service Children’s Nutrition Research Center, Departments of Pediatrics and Molecular and Human Genetics, Baylor College of Medicine, Houston, TX 77030, USA.

## Abstract

The molecular mechanisms responsible for the developmental origins of later disease are currently unknown. We previously demonstrated that women’s periconceptional nutrition predicts their offspring’s DNA methylation at metastable epialleles (MEs). We present a genome-wide screen yielding 687 MEs and track their trajectories across nine developmental stages in human in vitro fertilization embryos. MEs exhibit highly unusual methylation dynamics across the implantation-gastrulation transition, producing a large excess of intermediate methylation states, suggesting the potential for differential programming in response to external signals. Using a natural experiment in rural Gambia, we show that genomic regions sensitive to season of conception are highly enriched for MEs and show similar atypical methylation patterns. MEs are enriched for proximal enhancers and transcription start sites and are influenced by genotype. Together, these observations position MEs as distinctive epigenomic features programmed in the early embryo, sensitive to genetic and periconceptional environment, and with the potential to influence phenotype.

## Teaser

We have tracked DNA methylation dynamics at metastable epialleles across multiple developmental stages in the early human embryo.

## INTRODUCTION

DNA methylation is a widely studied epigenetic mark that plays a key role in the transcriptional regulation of a number of cellular processes in mammals including cell differentiation, genomic imprinting, and X-inactivation (*1*). Early embryonic development represents a critical window for the establishment of the methylome, when the human preimplantation embryo undergoes substantial remodeling, with widespread erasure of gametic methylation marks as the embryo transitions to a pluripotent state (*1*–*3*). Animal and human studies indicate that the methylation state can be influenced by the environment of the early embryo, suggesting a potential role for methylation and related epigenetic marks in mediating the effects of early-life nutritional and other environmental stressors on later health and disease (*4*–*6*).

Metastable epialleles (MEs) are genomic regions that show systemic (cross-tissue) interindividual variation in methylation, indicating establishment of the variable methylation state in the preimplantation embryo, before gastrulation (*7*). Murine and human MEs have been associated with the presence of neighboring transposable elements and are influenced by nutritional and other environmental stressors at periconception (*8*–*13*). Variable methylation at MEs was originally defined as occurring in the absence of genetic variation (*7*), and MEs have been widely studied in isogenic mice (*14*–*16*), demonstrating that systemic, stochastic variation in methylation state can occur independent of genotype. As we expand the search for ME regions into genetically heterogeneous human populations, we suggest that this definition should be extended to include genomic regions whose epigenetic state is under partial but nondeterministic genetic influence.

## RESULTS

### Screen for human MEs

**Fig. 1:**
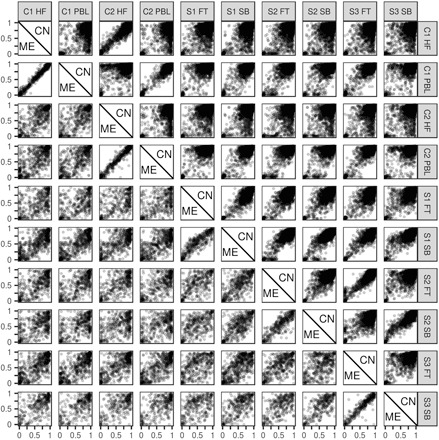
Methylation of ME and control regions in samples used for the ME screen. Methylation distributions in 687 ME and 5902 control regions. *X* axes represent methylation in the sample given by the label at the top of the column. *Y* axes, the same, but given by label at the end of the row. Each point represents mean methylation across all CpGs in a single region. Samples are listed by individual and tissue. HF, hair follicle; PBL, peripheral blood lymphocytes; FT, fat; SB, small bowel. Methylation in MEs is on the bottom (left) of the diagonal; methylation of controls (C) is on the top (right).

We previously reported the first-in-human genome-wide screen for MEs using whole-genome bisulfite-seq (WGBS) data from two tissues in two North American Caucasian individuals (*8*). This screen was limited by its inclusion of only two germ layer lineages (mesoderm and ectoderm) and its small sample size. For the current analysis, we produced an updated list of putative human ME regions by extending the previous ME screen to include samples from three new individuals and from an endodermal tissue (*17*). These 687 ME regions exhibit systemic interindividual variation in DNA methylation that is distinct from the patterns in 5902 genomic clustered “control” regions generated from the whole-genome background using the same clustering parameters used to find the ME regions (Fig. 1, table S1, fig. S1A, and Materials and Methods).

**Fig. 2:**
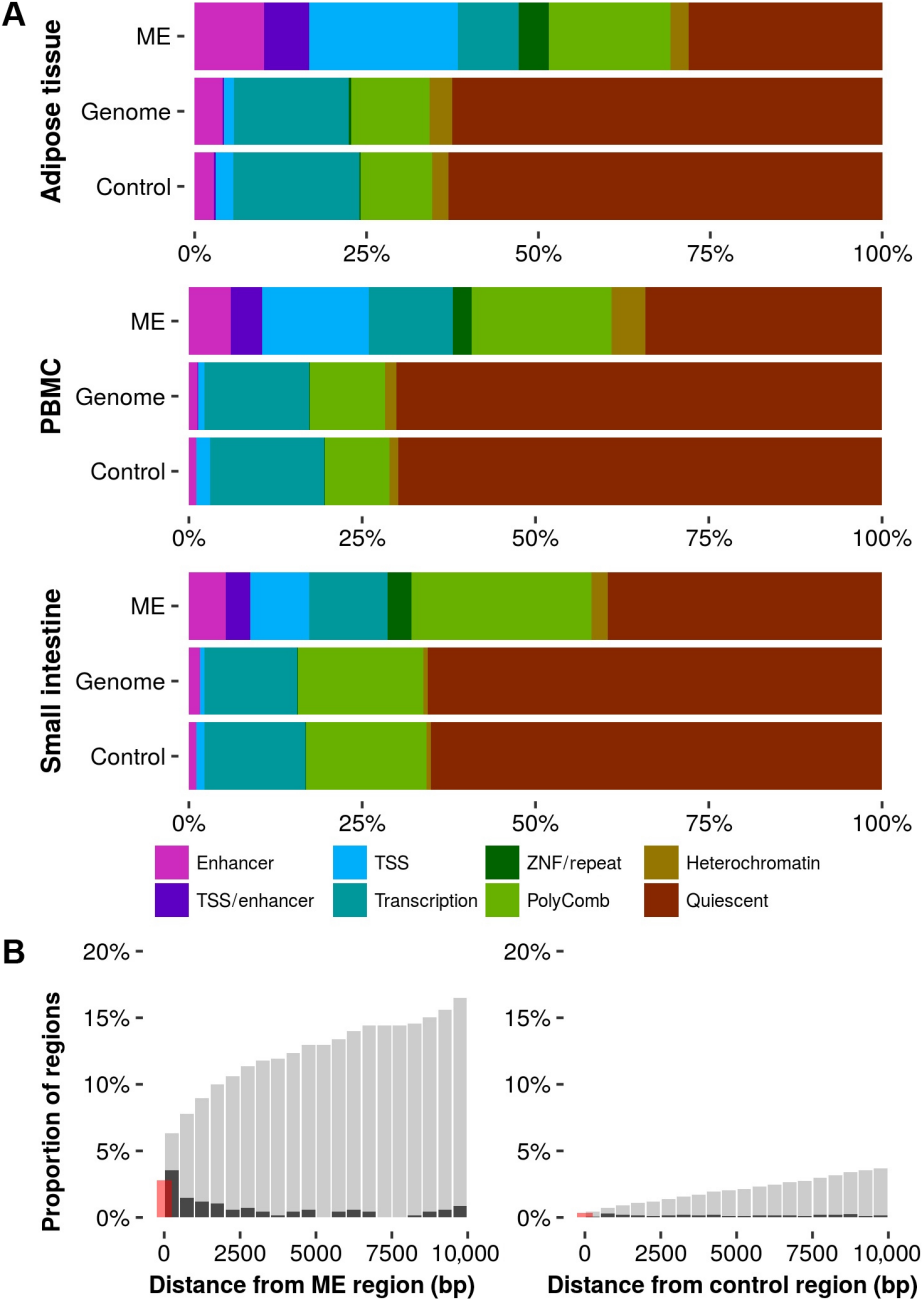
Characteristics of ME and control regions. (**A**) Proportion of MEs, control regions, and the whole genome, overlapping predicted chromatin states from three Roadmap samples (one each from endoderm, mesoderm, and ectoderm). Fifteen chromHMM states have been reduced to eight after combining states, which represent similar genomic features. Quiescent loci are those with low signal in all of the histone marks used as input to the chromHMM algorithm; ZNF regions are those overlapping zinc finger protein–encoding genes. (**B**) Proximity of MEs and controls to ZFP57 binding sites. Black bars show the proportion of regions with a binding site within the distance marked on the *x* axis. Gray bars show the cumulative proportion of regions within a given distance of a binding site. Red bars show the proportion of regions that overlap a binding site. PBMC, peripheral blood mononuclear cell; TSS, transcription start site.

Chromatin state at MEs was assessed using the histone-based 15-state chromHMM model (*18*) in three adult tissues (one derived from each germ layer), generated as part of the Roadmap Epigenomics Project (*19*). We observed that ME regions are more likely to be associated with predicted enhancers, transcription start sites, and zinc finger genes, and less likely to be associated with transcribed regions or regions with low levels of histone marks, compared to control regions and to genomic background in all three tissues surveyed (Fig. 2A). Furthermore, MEs are more likely to be near certain classes of transposable elements than would be expected given the genomic distribution of CG dinucleotides (CpGs), with significant enrichment for proximity to endogenous retroviral sequence 1 (ERV1) and endogenous retrovirus group K (ERVK) elements [*P* = 4.7 × 10^−8^, *P* = 1.4 × 10^−14^, Fisher’s exact test (FET); see Materials and Methods and table S2]. This is in line with our previous observations (*8*) and with observations in mice (*15*).

### Methylation dynamics of MEs during embryonic development

Patterns of systemic interindividual variation at MEs (Fig. 1) suggest that the methylation state is established in the early embryo, prior to separation into germ layers at gastrulation (*7*), but this has not yet been demonstrated in human embryonic samples. We analyzed human methylomes from early developmental stages in Chinese embryos using public data (*20*). This data set consists of reduced representation bisulfite-seq (RRBS) data from two to four biological replicates at each of nine developmental stages, from gametes, through cleavage-stage embryos, to blastocyst and differentiated embryonic tissue (table S3). More than 3.5 million CpG sites were covered in at least one of the replicates (fig. S1B), and despite RRBS covering only approximately 10% of genome-wide CpGs, a substantial proportion (44%) of our ME regions were covered in the Guo *et al*. data set (table S4).

**Fig. 3:**
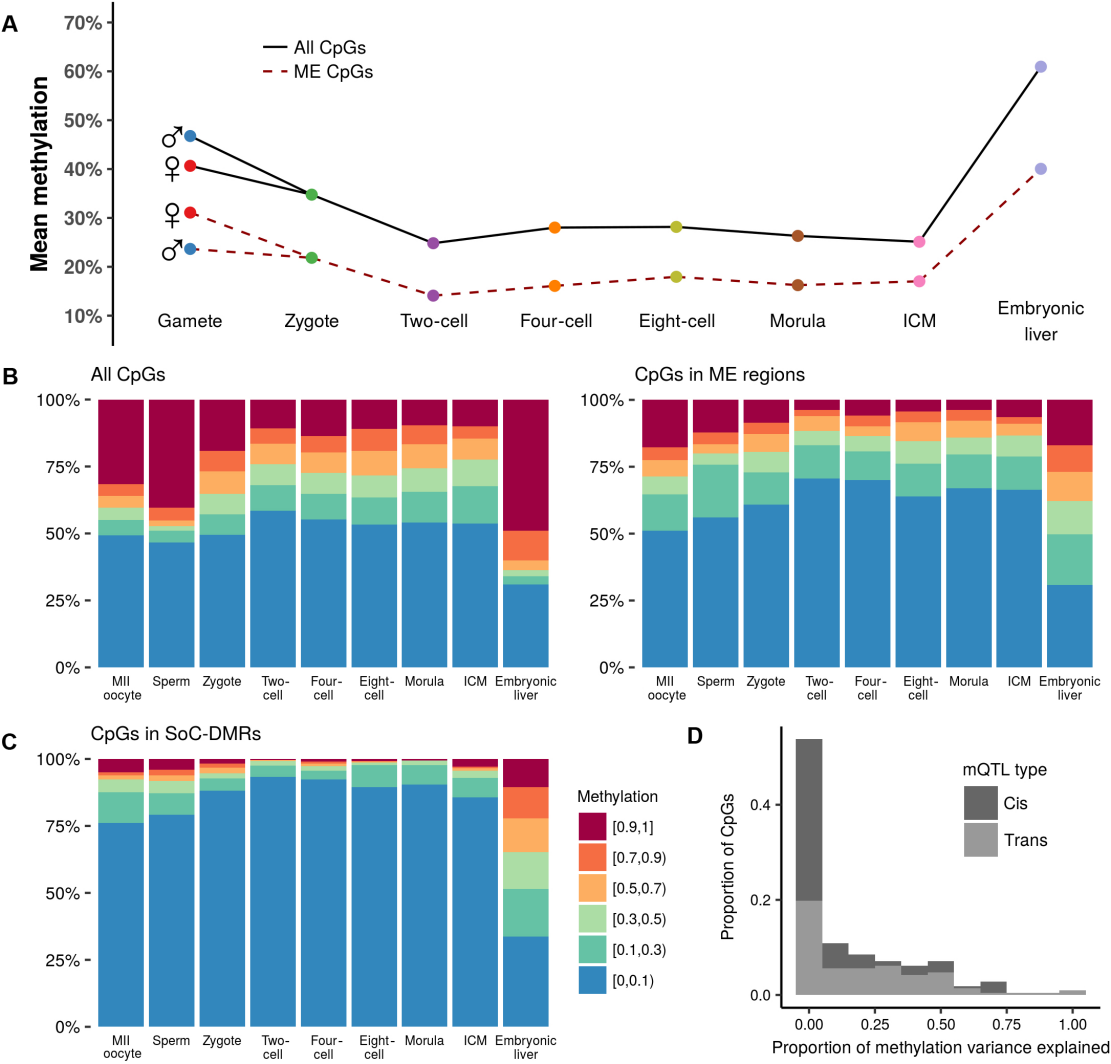
Methylation at MEs, all-RRBS background, and SoC-associated loci, and potential genetic influences. Within each developmental stage in all plots, CpGs are counted once for each replicate for which there was sufficient read depth. (**A**) Mean methylation at each developmental stage assayed by Guo *et al*. Solid line: mean methylation in all-RRBS background (*n* = 3,679,155 CpGs); dashed line: mean methylation at ME regions covered by RRBS (*n* = 302 regions; 2098 CpGs). (**B**) Distribution of methylation across developmental stages in all-RRBS background CpGs (left) and at CpGs within ME regions (right). See legend in (**C**). (C) Distribution of methylation at each stage in previously identified season of conception–associated differentially methylated regions (SoC-DMRs; see Materials and Methods). (**D**) Proportion of methylation variance explained by genetic variants in cis and in trans at ME-CpGs. These data are obtained from a previous comprehensive screen for methylation quantitative loci (mQTL)–CpGs in a large European study (*23*).

As a first step, we replicated the analysis from Guo *et al*. (*20*) showing genome-wide changes in mean methylation at each developmental stage (Fig. 3A). Expected patterns of genome-wide demethylation and remethylation at periconception and across the gastrulation transition are visible in this data set. In contrast to genomic background, methylation at MEs is consistently lower at all developmental stages, particularly in sperm (Fig. 3A). Mean methylation in clustered control regions is higher than in genomic background, making the contrast with ME methylation even greater (fig. S2). Similar patterns are evident in a mostly single cell–derived data set from the same group (fig. S3 and Materials and Methods) (*21*). Since change in global mean methylation is a relatively crude measure of methylation dynamics in the early embryo, we next considered the distribution of methylation at each stage (Fig. 3B). As suggested by Fig. 3A, we observed an increased proportion of low methylation states in ME regions relative to background (that is, all CpGs covered in the data set).

**Fig. 4:**
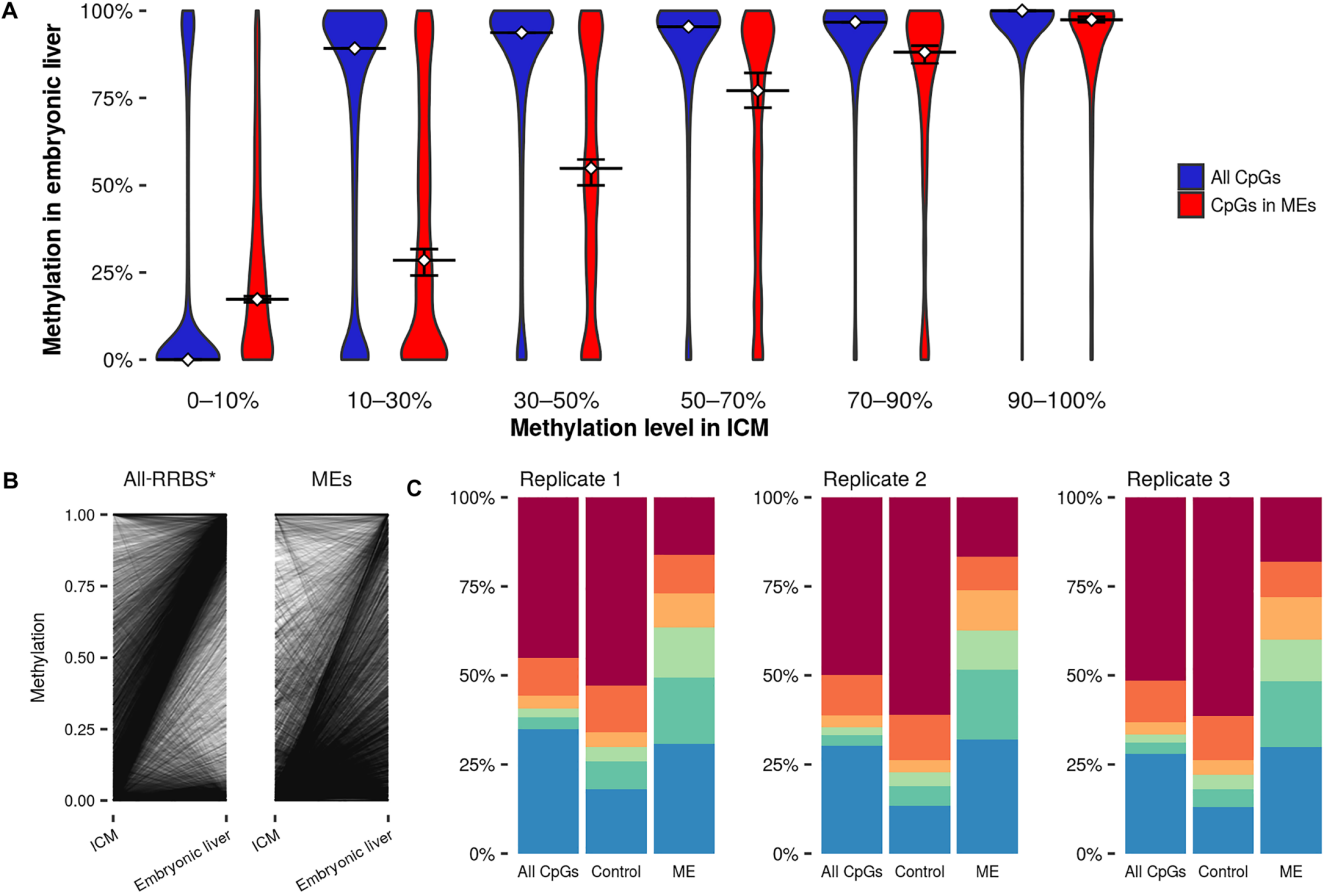
Methylation dynamics around the gastrulation transition. (**A**) Distribution of CpG methylation in embryonic liver, stratified by methylation level in ICM. Horizontal lines show the median methylation in embryonic liver. Error bars represent 95% bootstrapped confidence intervals. (**B**) Change in methylation at individual CpGs across the transition. Each line represents the change in methylation at a CpG for a single combination of replicates from each stage. *Because of the large number of data points, “all-RRBS” plot shows a random 0.1% sample of all possible ICM–embryonic liver transitions. (**C**) Distribution of methylation states in each embryonic liver replicate.

Most striking, however, was a marked increase in intermediate methylation states (sites with 10 to 90% methylation) within ME regions in the post-implantation embryo (“embryonic liver”) (Fig. 3B). Here, 52.3% of CpGs in ME regions show intermediate methylation versus 20.1% in genomic background and 26.6% in control regions (*P* < 2.2^−16^, chi-squared test for difference in proportions for both comparisons; fig. S5B). Genome-wide, there was a tendency toward a bimodal distribution of very high or low methylation after gastrulation (Fig. 4, A and B, and fig. S4). In contrast, in ME regions, a substantial proportion of CpGs are intermediately methylated in post-gastrulation tissue, irrespective of starting methylation state in (pre-gastrulation) inner cell mass (ICM) tissue. These distinctive patterns are evident in other fetal tissues (intestine, kidney, and lung) obtained from the Roadmap Epigenomics Project (*19*) and are also evident when comparing MEs with clustered control regions (fig. S5, A and B).

**Fig. 5:**
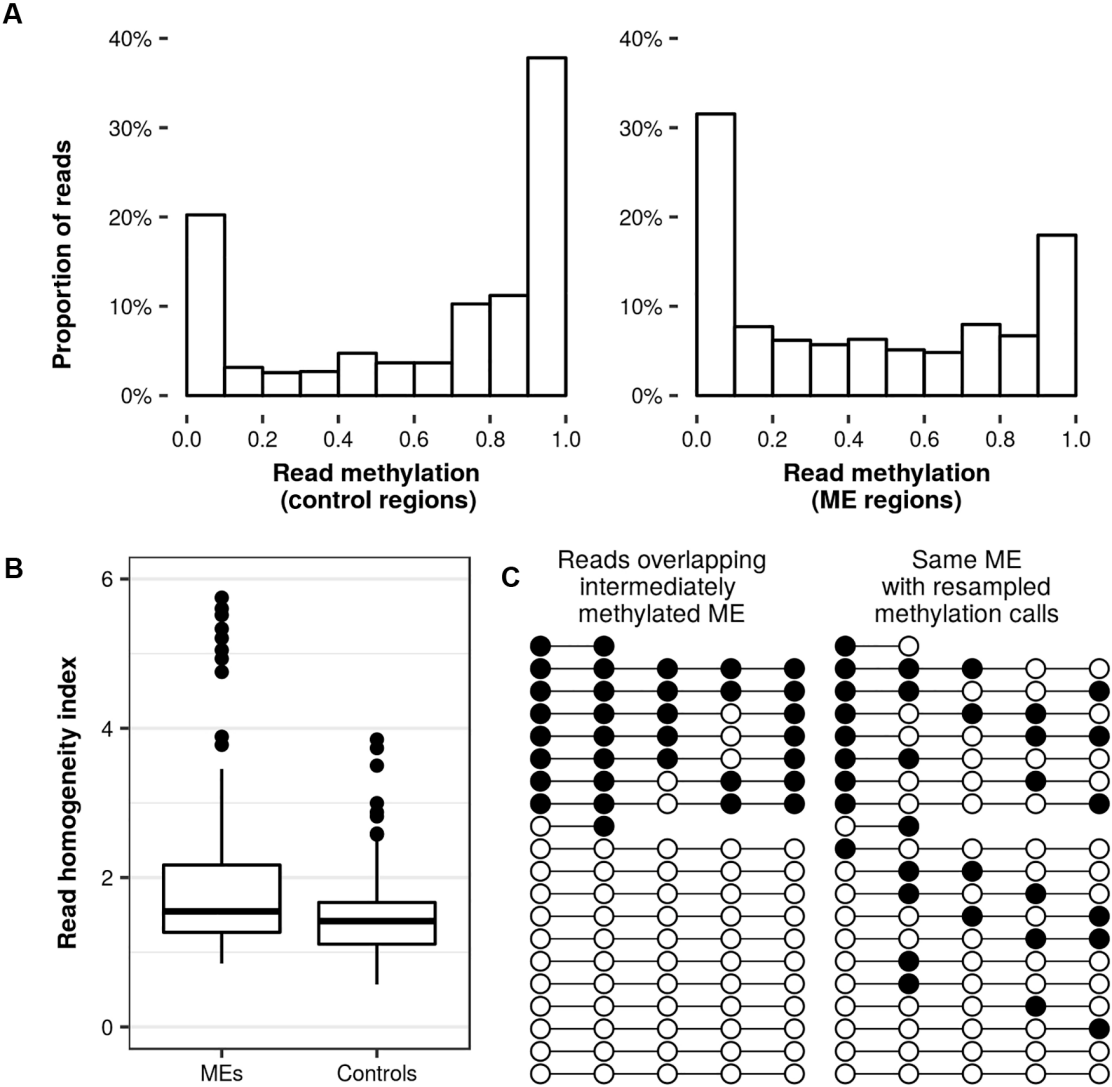
Read-level methylation analysis. (**A**) Distribution of read-level methylation for reads overlapping CpGs with intermediate methylation (10 to 90%) in clustered control regions (left, 31.8K reads) and ME regions (right, 22.5K reads). (**B**) RHI scores for MEs (*n* = 133) and control regions (*n* = 200) covered in the Guo *et al*. data show a significantly higher average RHI in MEs (Mann-Whitney, *P* < 5 × 10^−5^; nine MEs and two control outliers with RHI > 6 not shown). (**C**) Lollipop plots showing methylation calls overlapping an ME (left, reads ordered by read-level methylation) and a random resampling of the methylation calls, preserving the location of the reads (right). At this ME, the observed methylation calls result in a transition count of 11, while the resampled example has a transition count of 34, which is the median of 1000 resamplings performed on this region. The RHI of this region is thus 34/11 or 3.1.

Intermediate methylation states in pooled samples may result from interindividual variation with low intraindividual variation, heterogeneity of cell methylation states within individuals, or a combination of both. In the Guo *et al*. data set, most replicates at each developmental stage consist of cells derived from several embryos (table S3). However, each embryonic liver replicate represents only one conceptus, allowing us to assess the methylation profile of three individuals separately. In each of these replicates, we still observe a large proportion of intermediate methylation states in ME regions (Fig. 4C), indicating that intermediate methylation is driven by intratissue heterogeneity of methylation states within an individual, rather than by interindividual differences. A read-level analysis suggests that intermediate methylation at the CpG level arises from a combination of partially (10 to 90%) and fully methylated/unmethylated (molecule-specific) reads, within both ME and clustered control regions (Fig. 5A).

To test this possibility, we devised a read homogeneity index (RHI) to evaluate the extent to which intermediately methylated clusters are driven by mixtures of reads each with high methylation homogeneity. This normalized index accounts for differences due to cluster size and average methylation (see Materials and Methods). Clusters containing more reads with long runs of methylated or unmethylated CpGs will have a higher RHI (see Fig. 5C for a representative illustration). We found that intermediately methylated ME clusters have a significantly higher RHI than equivalent control clusters (Mann-Whitney, *P* < 3 × 10^−5^; Fig. 5B), indicating increased levels of molecule-specific methylation at MEs.

### Association of genetic variation and proximal protein binding sites with ME methylation

The above observations in single replicates indicate that cellular differences in methylation state occur independent of genetic variation. It remains the case, however, that methylation at MEs might be influenced by genotype (*22*). Our ME screen filtered out differences in methylation for CpGs within 60 base pairs (bp) of a genetic variant (see Materials and Methods), but observed interindividual differences may be driven by more distant genetic variation. The identification of mQTL—genetic variants influencing CpG methylation—requires large sample sizes, and so far, sufficiently powered studies have only been conducted using methylation arrays that assay a small fraction of the methylome. We therefore assessed potential genetic influence on DNA methylation at MEs identified in our WGBS screen by analyzing mQTL identified in a large UK study (see Materials and Methods) (*23*). This analysis identified genetic influences from 8.3 million common single-nucleotide polymorphisms (SNPs) on approximately 400,000 CpGs assayed in 3948 blood samples taken at five time points. We observed highly significant enrichment for mQTL at CpGs in ME regions [odds ratio (OR), 6.2; *P* < 10^−10^, FET] but not in control regions (OR, 1.3; *P* = 0.09). Estimates of the proportion of methylation variance explained by cis and trans mQTL at MEs indicate that mQTL explain only a small to moderate proportion of methylation variance at mQTL-associated ME-CpGs {median proportion of variance explained, 0.23 [interquartile range (IQR), 0.057 to 0.46] in birth samples and 0.24 (IQR, 0.055 to 0.49) in adult samples; Fig. 3D and Materials and Methods}, suggesting that stochastic and/or environmental factors play a major role in driving the distinctive early embryo dynamics observed at MEs.

Control of methylation dynamics in the preimplantation embryo is critical in genomic imprinting (*24*). The Krüppel associated box (KRAB) zinc finger protein ZFP57 and its binding partner tripartite motif-containing 28 (TRIM28), along with the multi-zinc finger protein CCCTC-binding factor (CTCF), have been implicated in the maintenance of parental methylation marks in the early embryo (*25*–*27*). We therefore speculated that these might influence methylation dynamics at MEs. Using public human chromatin immunoprecipitation sequencing (ChIP-seq) data, we found significant enrichment for proximal ZFP57, CTCF, and TRIM28 binding sites in ME regions compared to genomic background (respectively *P* < 2.2 × 10^−16^, *P* < 2.2 × 10^−16^, *P* = 4.1×10^−7^, FET; table S2 and Materials and Methods). For example, 16.3% of ME regions are within 10 kb of a ZFP57 binding site, compared with 3.7% of clustered control regions (7.8% versus 0.7% using a 1-kb distance threshold; 2.8% versus 0.3% using exact overlaps; Fig. 2B and table S1).

To investigate the potential influence of ZFP57 binding on ME methylation, we analyzed data from a study of individuals homozygous for ZFP57 mutations associated with transient neonatal diabetes, a rare imprinting disorder (*28*). By comparing individuals with ZFP57 mutations with matched controls, this study found 61 DMRs (ZFP57m-DMRs), which were common to at least two patients with a homozygous mutation. Despite the small number of ZFP57m-DMRs, five overlapped an ME region and a further two were within 10 kb (table S5). All were hypomethylated in the individuals with the mutation. In contrast, no control clusters overlap or are within 10 kb of a ZFP57m-DMR.

## Influence of periconceptional environment on ME methylation

We previously reported associations between season of conception (SoC) and DNA methylation at a small number of MEs in a Gambian population (*8*, *29*, *30*). In this rural community in sub-Saharan Africa, seasonality is linked to significant differences in maternal diet and circulating maternal methyl donor biomarkers (*31*), suggesting that, as observed at murine MEs, human MEs may be sensitive to the periconceptional nutritional environment (*8*, *11*, *30*). We therefore hypothesized that Gambian SoC-associated genomic regions may show patterns of early embryo methylation dynamics similar to those observed at MEs from our WGBS screen in North American Caucasians.

To investigate this possibility, we curated a list of 21 SoC-DMRs previously identified in epigenome-wide association studies from two independent Gambian studies (see Materials and Methods). Gambian SoC-DMRs show extremely low levels of methylation in the Chinese gametes and pre-gastrulation embryonic tissues assayed by Guo *et al*., with strong enrichment of intermediate methylation states relative to background in the post-gastrulation embryo (*n* = 17 SoC-DMR regions with ≥1 CpG in the Guo *et al*. data set; Fig. 3C), reminiscent of patterns observed at MEs (Fig. 3B). SoC-DMRs were highly enriched for overlapping MEs (5 of 21 DMRs; *P* < 2.2 × 10^−16^, FET) and for mQTL (*P* = 7.1 × 10^−16^, FET).

### DISCUSSION

We have presented the first-in-human characterization of methylation dynamics at MEs in early embryos. Our data suggest that the gastrulation transition is key to the establishment of intermediate methylation states at MEs and that these states are driven by intratissue variegation effects, as originally proposed (*7*). In addition, our analysis suggests that interindividual variation at MEs is influenced by at least three factors: stochastic or probabilistic processes, periconceptional environmental exposures, and genomic context. The last of these is notable since previous studies of murine MEs have generally considered isogenic populations, so epigenetic metastability in humans was assumed to be definitively free of genetic effects. We note, however, that SNP heritability estimates from array-based mQTL studies necessarily cover relatively few ME-CpGs in our WGBS screen, so further work is required to better understand the influence of genotype on ME methylation.

Our work confirms previous findings in humans and in mice of a link between metastability and proximal transposable elements and further indicates a potential role for zinc finger proteins including CTCF and ZFP57. Recent work characterizing haplotype-dependent allele-specific methylation (hap-ASM) in humans has identified polymorphic CTCF binding sites as important drivers of hap-ASM including at ZFP57, indicating that CpG methylation at this locus is at least partially dependent on haplotype (*32*). Van Baak *et al*. (*33*) recently observed interindividual variation of methylation between individuals sharing the same haplotype at *ZFP57*, indicating a potential role for stochastic processes and environmental influence in the context of genetic effects, as we observe at MEs in embryonic liver tissue from single embryos. As MEs are frequently located near ZFP57 binding sites, altered expression of ZFP57 due to variable methylation near its transcription start site could contribute to the variance in methylation at MEs. This notion is supported by our finding that several ZFP57 binding site–proximal MEs are among DMRs associated with human ZFP57 mutations.

Chromatin state analysis suggests that, compared to background, MEs tend to be located within enhancers and proximal to transcription start sites, indicating potential effects on transcriptional regulation. While further work is required to link methylation status to gene expression, this analysis positions MEs as possible mediators of phenotypic plasticity in response to periconceptional exposures, as has been proposed in previous models of developmental programming (*34*). The presence of variably methylated sites that are linked to gene function, and are susceptible to environmental and genetic influence, has been proposed as a potential adaptive mechanism (*35*), positioning MEs as prime candidates for investigating adaptive responses to changing environments.

## MATERIALS AND METHODS

### ME screen using WGBS data

Two WGBS data sets were used to search for human MEs: that used in our previous screen (two Caucasian adult males, two tissues: peripheral blood and hair follicle) (*8*) and an additional data set with three individuals in the Roadmap Epigenomics Project (S1: male, 3 years old, Caucasian/African-American; S2: female, 30 years old, Caucasian; S3: male, 34 years old, Caucasian; two tissues: small bowel and fat) (*19*). Bismark v0.14 (*36*) was used to map the raw reads and extract methylation at CpGs. In each sample, CpGs with less than 10 times the read depth were filtered out. ME regions were generated using a three-step process: (i) identify individual CpGs showing the hallmarks of metastability (“ME-CpGs”) in each data set, (ii) run a clustering algorithm on these loci, and (iii) filter out clusters that contain many non–ME-CpGs. For the first step, CpGs were required to have at least a 15% absolute difference in methylation between individuals and an absolute intertissue methylation difference no greater than one-third of the absolute interindividual difference. This 15% methylation difference was assessed separately for the two WGBS data sets. Within each of these two data sets, SNPs were called in each individual from the bisulfite-converted reads using bis-SNP (*37*). As a precaution against overselecting for methylation variation driven by proximal variants (for example, CpGs with ASM), any CpG within 60 bp of a SNP (*22*) was excluded from consideration in the data set containing the SNP. ME clusters were generated by filtering out ME-CpGs that were not within 300 bp of another ME-CpG, and then requiring that each resulting cluster had four or more ME-CpGs. The final list of 687 ME regions was then filtered from those clusters by selecting only those which had at least twice as many ME-CpGs as non–ME-CpGs.

### Control cluster generation

We first considered deriving a set of control regions as those showing interindividual variation without intertissue concordance; however, few such regions exist in our data sets. We therefore chose regions by applying clustering parameters identical to those used for ME clusters to all genomic CpGs covered in our WGBS data sets, irrespective of methylation. A sample of the resulting regions, matching the joint distributions of region size and number of CpGs per region found in the MEs, was then used to generate 5902 control regions (fig. S1A).

### Enrichment for proximal genomic features

Transposable element regions [LINE (long interspersed nuclear elements), SINE (short interspersed nuclear elements), and LTR (long terminal repeats)] of the human genome (hg19), determined by RepeatMasker, were downloaded from the UCSC (University of California, Santa Cruz) ( hg19 annotations repository. Human cell line ChIP-seq binding sites were downloaded from the GEO (Gene Expression Omnibus) or ENCODE (Encyclopedia of DNA Elements) [ZFP57: human embryonic kidney 293T (HEK293T) cells, GSM2466450; TRIM28: HEK293T cells, ENCFF002CRN; CTCF: H1–hESC (human embryonic stem cells), ENCFF002CDS]. For enrichment testing, the genome was divided into 1000-bp “bins” or “tiles” (*8*, *20*), and the number of tiles overlapping a feature of interest was compared with the number of tiles overlapping features within 10 kb of an ME. FET was then used to determine the OR and *P* value for enrichment of ME-proximal features. The list of *ZFP57*-mutant DMRs was from Bak *et al*. (*28*) (table S5).

### Guo *et al.* and Zhu *et al.* data analysis

RRBS methylation data from all replicates of each of the stages analyzed were downloaded from GEO (accession number GSE49828). Sites were considered covered if total read depth from both strands was at least 10. CpG coverage within each sample and overall is summarized in fig. S1B and table S3. For the read-level analyses in the embryonic liver samples, raw reads were downloaded from the Sequence Read Archive (SRA). Reads were trimmed and filtered for quality and size by Trim Galore! and mapped by Bismark v0.18 (*36*), without deduplication, as recommended by the Bismark authors for RRBS data. Reads were further filtered to exclude those containing fewer than four CpGs. Read-level methylation was then determined by taking the average of all CpGs included in the read (that is, the number of methylated CpGs divided by the total number of CpGs present on the read). Only CpGs with 10 times the coverage were considered when computing CpG-level methylation. CpGs falling outside the ME or control regions were not analyzed, and CpGs with >200 times the coverage were ignored to prevent highly overrepresented regions from dominating the analysis. Bootstrapped confidence intervals for medians in Fig. 3A were generated by resampling the original data 1000 times.

Data from Zhu *et al*. (*21*) were downloaded from GEO (accession number GSE81233), including all single-cell and bulk cell data sets from MII oocyte, sperm, zygote, two-cell, four-cell, eight-cell, morula, ICM, and each of the three embryonic heart samples. All data were gathered by developmental time point, and all CpGs with read depth ≥10 were included in the plots of developmental time point–level means. Because of the small number of samples at many developmental time points (compounded by the fact that single-cell reads only provide two molecules’ worth of information), no further analysis on these samples was performed.

### Read homogeneity index

For each ME and control cluster, all reads from the Guo *et al*. embryonic liver replicates overlapping the cluster were combined, and any CpGs outside of the cluster were ignored. We initially considered using a published method for this, such as methylation haplotype load (*38*), but developed the RHI instead as other methods assume a large number of reads overlapping a fixed set of adjacent CpGs, whereas the bisulfite-seq reads from Guo *et al*. sometimes contain uncalled cytosines (for example, due to SNPs or a low-quality base call) or only overlap the first or last one to two CpGs of an ME/control region (for example, the short reads in Fig. 5C). To account for the effects of differences in read and cluster size, and overall methylation within a cluster, a normalized RHI was calculated as follows. First, the observed number of methylation “transitions” was counted, a transition being any instance where two adjacent CpGs have different methylation calls. Next, the same measure was computed, this time with an equal number of methylated CpGs randomly distributed across reads within the cluster. This randomization was repeated 1000 times. Finally, the RHI was calculated as the median transition count across all 1000 randomizations, divided by the observed (empirical) transition count. The RHI thus provides a measure of the degree to which reads are consistently methylated or unmethylated, compared to reads where methylated CpGs are randomly distributed. Note that the RHI will generally be greater than 1 due to the tendency for methylation states at neighboring CpGs to be correlated.

### mQTL analysis

CpGs associated with mQTL on the Illumina Infinium HumanMethylation450 BeadChip were downloaded from www.mqtldb.org. These were identified using samples from children (cord blood, *n* = 771; childhood, *n* = 834; adolescence, *n* = 837) and their mothers (pregnancy, *n* = 764; middle age, *n* = 742). See Gaunt *et al*. for further details (*23*). For the current analysis, MEs were tested for enrichment of mQTL-associated CpGs at any time point, relative to “reliable” non–mQTL-associated CpGs on the 450K array [see Gaunt *et al*. (*23*)], using FET. Data on proportion of methylation variance explained by cis (±1 Mb of CpG probe) and trans (non-cis) mQTL (“SNP heritability” in Gaunt *et al*.) were obtained from one of the authors (see Acknowledgments). Figure 3D shows the distribution of variance explained for CpGs overlapping MEs in “middle age” samples, this being the closest time point to the samples that were used for the ME screen.

### Gambian SoC-DMRs

SoC-DMRs were identified by taking the top 10 ranking DMRs [by family-wise error rate (FWER) using the “bumphunting” method (*39*)] from two SoC-association analyses in independent samples conducted using the Illumina 450K array in (i) PBL from Gambian infants (*8*) and (ii) PBL from Gambian 2-year-olds (*40*). A further seven SoC-DMRs ranked in the top 50 (by FWER) in both data sets were also included. This resulted in a total of 21 distinct (nonoverlapping) SoC-DMRs. Information on ethical approvals for each of the data sets analyzed in this study can be found in the source publications cited above.

## Supplementary Material

http://advances.sciencemag.org/cgi/content/full/4/7/eaat2624/DC1

## SUPPLEMENTARY MATERIALS

Supplementary material for this article is available at http://advances.sciencemag.org/cgi/content/full/4/7/eaat2624/DC1 Fig. S1. ME and control region sizes, and Guo *et al*. methylome coverage. Fig. S2. Mean methylation at MEs and clustered control regions assayed by Guo *et al*. Fig. S3. Mean methylation at all CpGs and at MEs and clustered control regions assayed by Zhu *et al*. (*21*). Fig. S4. Methylation dynamics at the ICM–to–embryonic liver transition. Fig. S5. ME background comparisons in other fetal tissues, and methylation in control clusters. Table S1. MEs identified in genome-wide screen. Table S2. Enrichment of proximal genomic features in MEs. Table S3. Number of CpGs covered in each replicate of RRBS data from Guo *et al*. Table S4. Size of ME and control regions, and their coverage in Guo *et al*. RRBS data. Table S5. Overlap of Bak *et al*. (*28*) ZFP57-mutant DMRs with MEs.
