# Supplementary material for: Establishment of environmentally sensitive DNA methylation states in the very early human embryo
Source: Sci Adv. 2018 Jul 11;4(7):eaat2624. doi: 10.1126/sciadv.aat2624 (PMC6040841; doi:10.1126/sciadv.aat2624)
Supplement: http://advances.sciencemag.org/cgi/content/full/4/7/eaat2624/DC1 [file supp_4_7_eaat2624__index.html]

Science Advances | Science Advances

## Supplementary Materials

**The PDF file includes:**

- Fig. S1. ME and control region sizes, and Guo *et al*. methylome coverage.
- Fig. S2. Mean methylation at MEs and clustered control regions assayed by Guo *et al*.
- Fig. S3. Mean methylation at all CpGs and at MEs and clustered control regions assayed by Zhu *et al*. (*21*).
- Fig. S4. Methylation dynamics at the ICM–to–embryonic liver transition.
- Fig. S5. ME background comparisons in other fetal tissues, and methylation in control clusters.

Download PDF

**Other Supplementary Material for this manuscript includes the following:**

- Table S1 (Microsoft Excel format). MEs identified in genome-wide screen.
- Table S2 (Microsoft Excel format). Enrichment of proximal genomic features in MEs.
- Table S3 (Microsoft Excel format). Number of CpGs covered in each replicate of RRBS data from Guo *et al*.
- Table S4 (Microsoft Excel format). Size of ME and control regions, and their coverage in Guo *et al*. RRBS data.
- Table S5 (Microsoft Excel format). Overlap of Bak *et al*. (*28*) ZFP57-mutant DMRs with MEs.

Download Tables S1 to S5

**Files in this Data Supplement:**

- Adobe PDF - aat2624\_SM.pdf
