# Supplementary material for: Establishment of environmentally sensitive DNA methylation states in the very early human embryo
Source: Sci Adv. 2018 Jul 11;4(7):eaat2624. doi: 10.1126/sciadv.aat2624 (PMC6040841; doi:10.1126/sciadv.aat2624)
Supplement: http://advances.sciencemag.org/cgi/content/full/4/7/eaat2624/DC1 [file aat2624_SM.pdf]

Science Advances
AAAS

advances.sciencemag.org/cgi/content/full/4/7/eaat2624/DC1

Supplementary Materials for

Establishment of environmentally sensitive DNA methylation states in the very early human embryo

Noah J. Kessler, Robert A. Waterland, Andrew M. Prentice, Matt J. Silver*

*Corresponding author. Email: matt.silver@lshtm.ac.uk

Published 11 July 2018, *Sci. Adv.* **4**, eaat2624 (2018)
DOI: 10.1126/sciadv.aat2624

The PDF file includes:

Fig. S1. ME and control region sizes, and Guo *et al*. methylome coverage.
Fig. S2. Mean methylation at MEs and clustered control regions assayed by Guo *et al*.
Fig. S3. Mean methylation at all CpGs and at MEs and clustered control regions assayed by Zhu *et al*. (*21*).
Fig. S4. Methylation dynamics at the ICM–to–embryonic liver transition.
Fig. S5. ME background comparisons in other fetal tissues, and methylation in control clusters.

Other Supplementary Material for this manuscript includes the following:

(available at advances.sciencemag.org/cgi/content/full/4/7/eaat2624/DC1)

Table S1. MEs identified in genome-wide screen.
Table S2. Enrichment of proximal genomic features in MEs.
Table S3. Number of CpGs covered in each replicate of RRBS data from Guo *et al*.
Table S4. Size of ME and control regions, and their coverage in Guo *et al*. RRBS data.
Table S5. Overlap of Bak *et al*. (*28*) ZFP57-mutant DMRs with MEs.

Supplementary Material

a

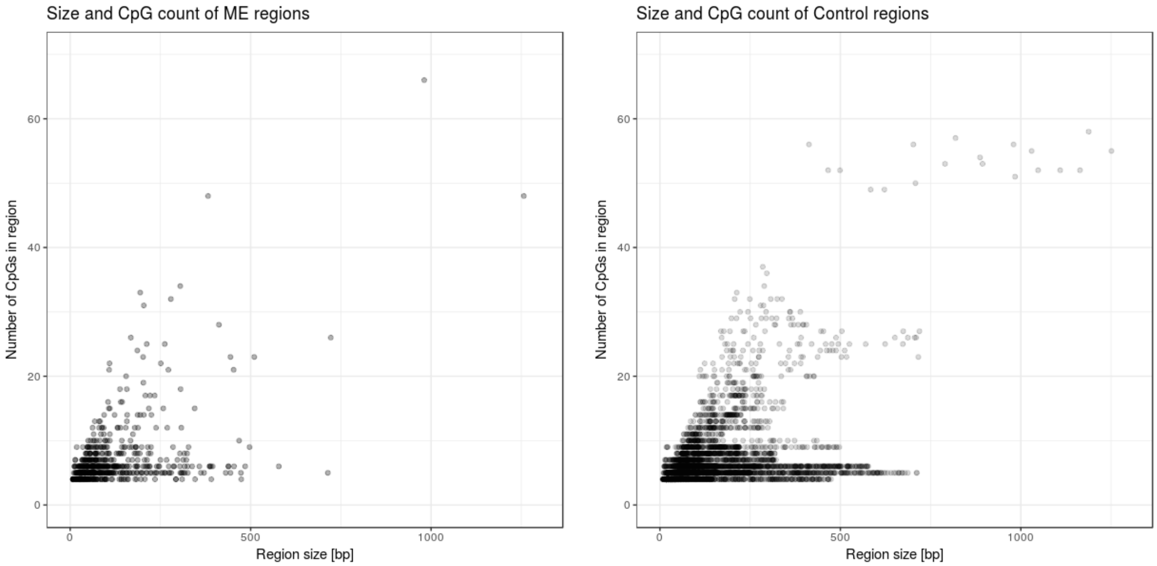

b

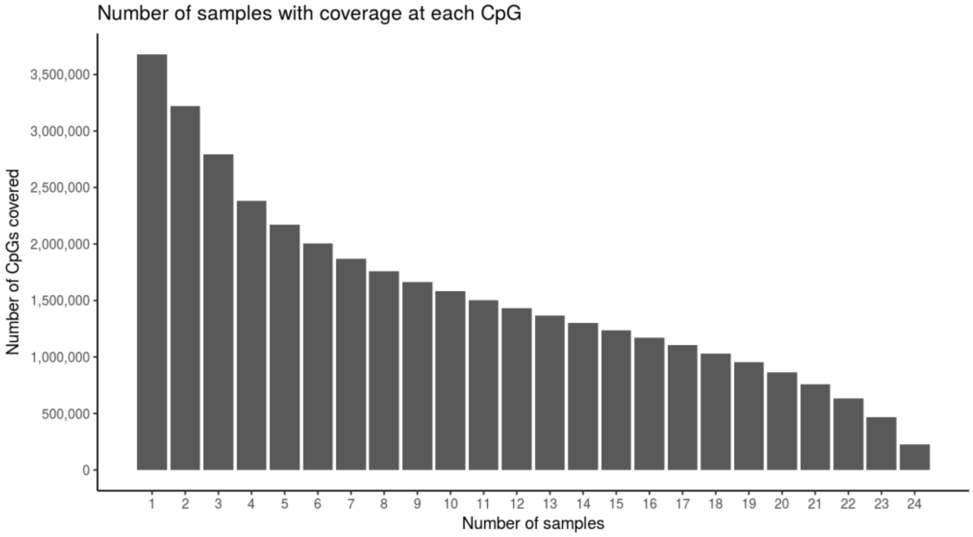

Fig. S1. ME and control region sizes, and Guo *et al*. methylome coverage. (**A**), The joint distribution of region size and number of CpGs in each region was approximated in creating the set of control regions. One outlier ME region of size >1500 bp is not shown. (**B**), Methylome coverage within Guo *et al*. samples. Bars represent the number of CpGs (y-axis) covered by at least the number of samples shown on the x-axis.

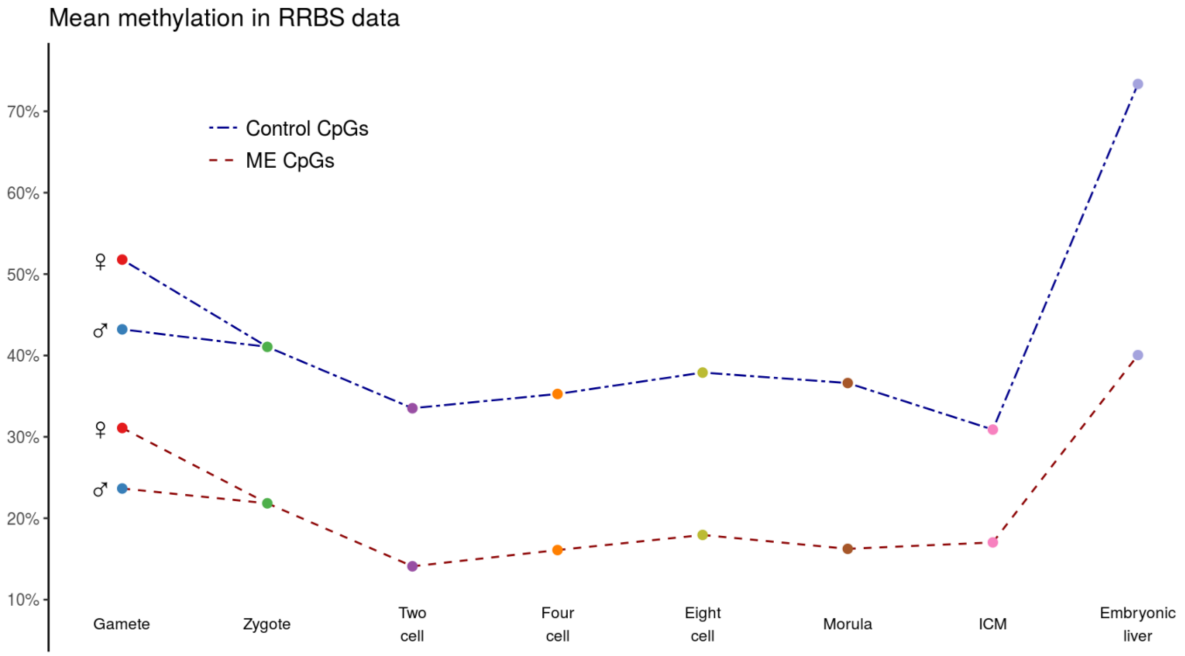

Fig. S2. Mean methylation at MEs and clustered control regions assayed by Guo *et al*. CpGs are counted once for each replicate for which there was sufficient read depth at each developmental stage. Dashed line: mean methylation in clustered control regions (n=1,184 regions; 6,975 CpGs); dashed line: mean methylation at ME regions covered by RRBS (n=302 regions; 2,098 CpGs).

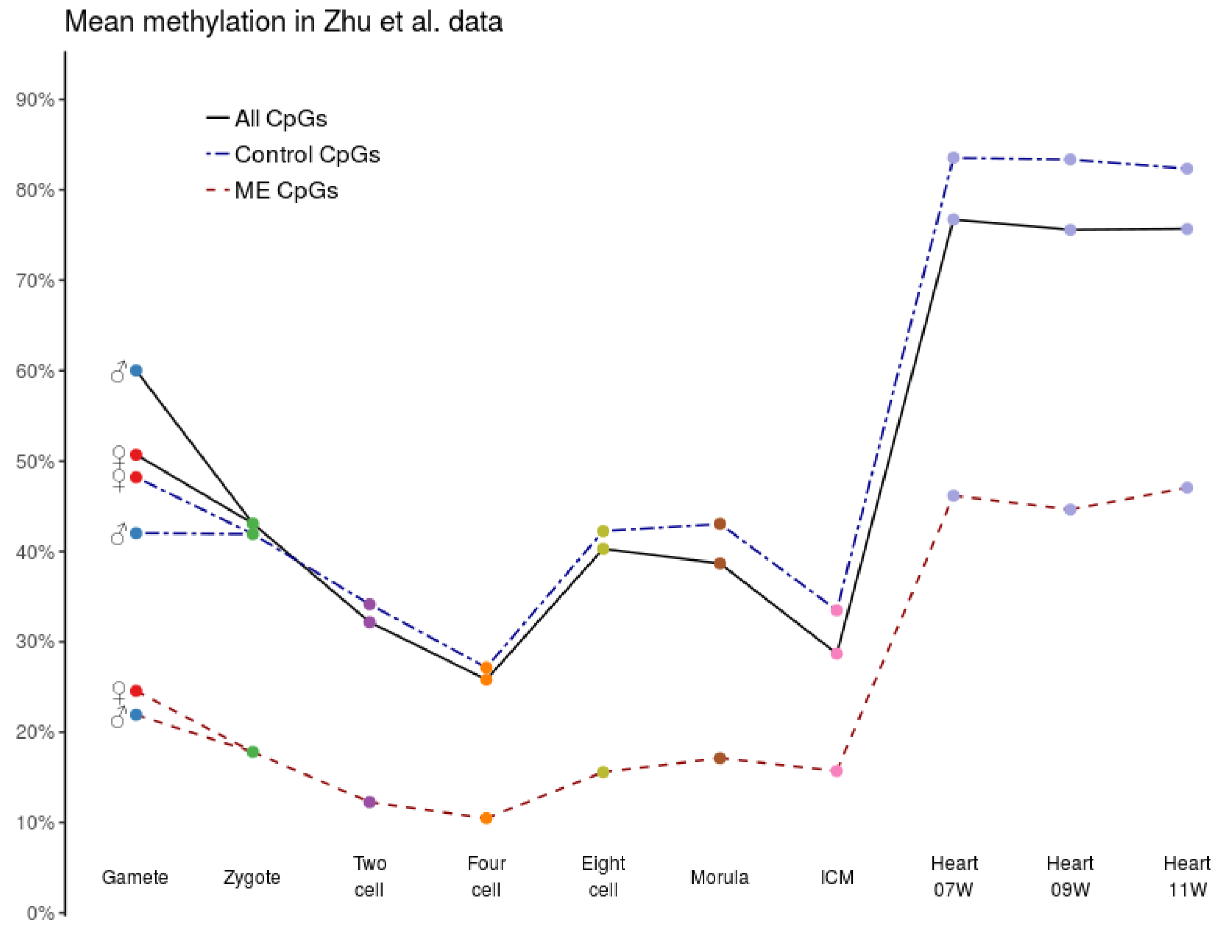

Fig. S3. Mean methylation at all CpGs and at MEs and clustered control regions assayed by Zhu *et al*. (*21*). Each CpG is counted once regardless of the number of samples/replicates which had reads overlapping it. Maximum number of CpGs with 10x coverage at any time point: 26,359,279 (All CpGs), 4,969 (MEs), 38,120 (Controls).

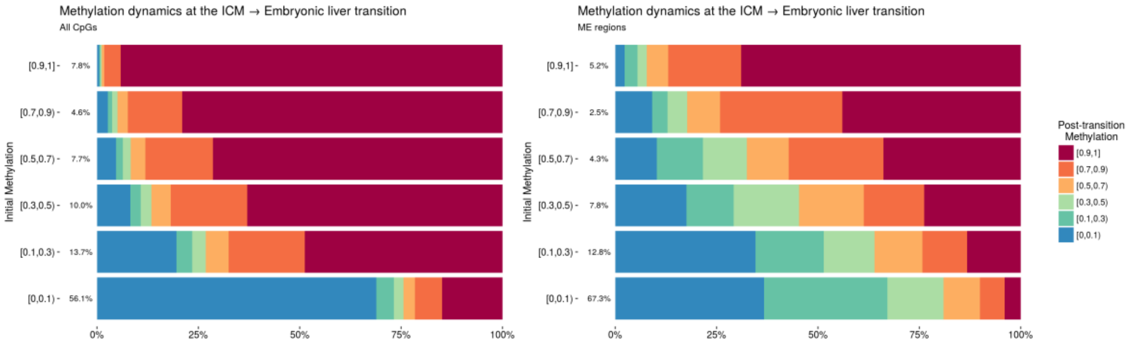

Fig. S4. Methylation dynamics at the ICM–to–embryonic liver transition. a, Same data as Figure 3, but in a two-dimensional format. The y-axis represents the methylation level in ICM. The proportion of CpGs within each of the six methylation ranges in ICM is given next to the bars. The x-axis represents the distribution of ending (embryonic liver) methylation of all sites from each starting methylation category.

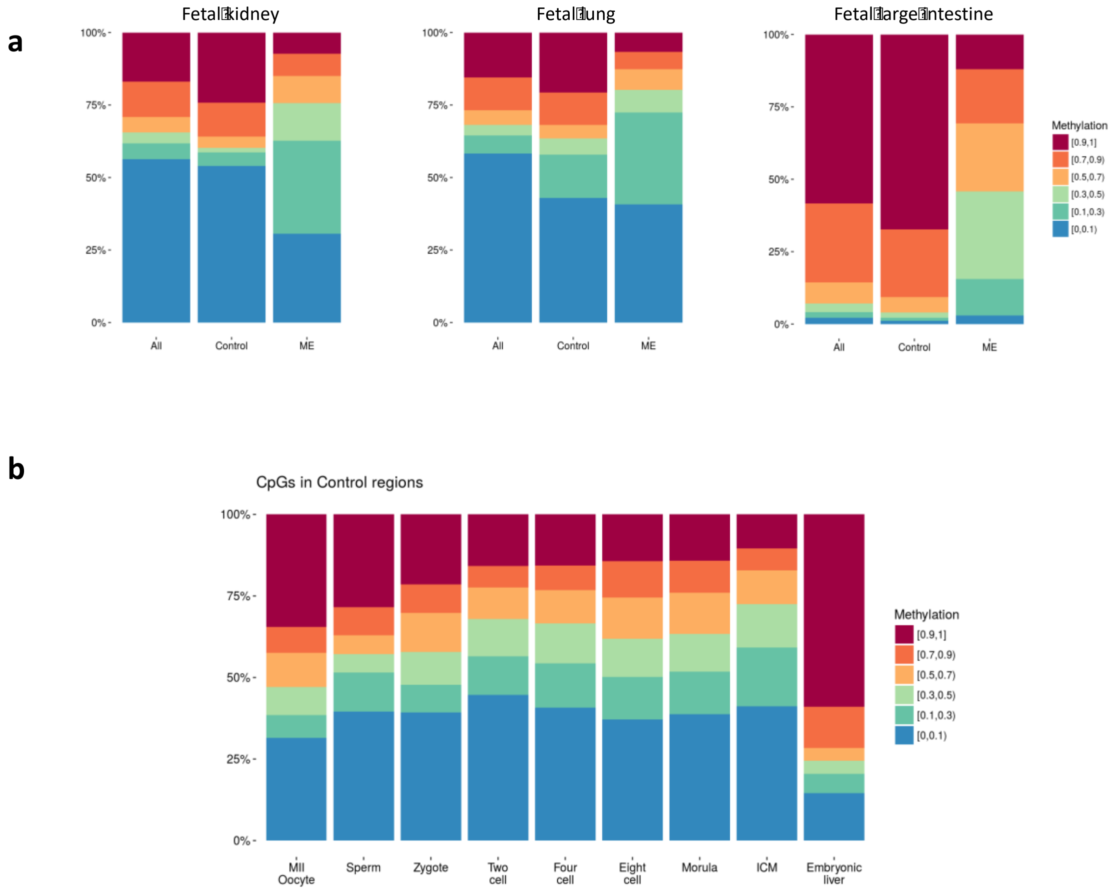

Fig. S5. ME background comparisons in other fetal tissues, and methylation in control clusters. (**A**), Methylation distribution in all CpGs, control regions, and ME regions in Roadmap fetal tissues: kidney (E086, RRBS); lung (E088, RRBS); and large intestine (E084, WGBS). Note that the WGBS sample has a much different distribution of methylation in genomic background compared to the RRBS datasets, but still shows an extreme enrichment for intermediate methylation in ME regions. (**B**), Distribution of methylation within control regions.
